# The Wisconsin Longitudinal Study: Overview, Data Linkages, and Future Plans

**DOI:** 10.1093/geroni/igab046.850

**Published:** 2021-12-17

**Authors:** Michal Engelman

**Affiliations:** University of Wisconsin-Madison, Madison, Wisconsin, United States

## Abstract

The WLS is a study of Wisconsin high school class of 1957 graduates, with follow-ups in 1964, 1975, 1993, 2004, 2011, and 2020. The data reflect the life course of the graduates (and their siblings), initially covering education, switching to family, career, and social participation in midlife, and physical and mental health, cognitive status, caregiving, and social support as respondents age. The WLS is linked to multiple administrative data sources including: parent earnings from state tax records (1957-60) and Social Security earnings and benefits for respondents; 1940 Census data; characteristics of high schools and colleges, employers, industries, and communities of residence; voting records from 2000-2018; Medicare claims; and the National Death Index. Efforts are underway to expand the racial/ethnic and educational composition of the WLS by supplementing the original sample with a new cohort of age-matched adults drawn from Wisconsin’s Black, Hispanic, Asian-American, and Native American communities.

